# A Case of Latent Plasmodium Falciparum Malaria in a Patient With Coexisting Systemic Lupus Erythematosus (SLE) and Neuromyelitis Optica Spectrum Disorder (NMOSD)

**DOI:** 10.7759/cureus.30436

**Published:** 2022-10-18

**Authors:** Ana Valle, Nick C Yu, Vasiliki Giannakakos, Rohan Maini, Matthew Shaines

**Affiliations:** 1 Internal Medicine, Albert Einstein College of Medicine, Bronx, USA; 2 Ophthalmology, Albert Einstein College of Medicine, Bronx, USA; 3 Hospital Medicine, Albert Einstein College of Medicine, Bronx, USA

**Keywords:** neuromyelitis optica, sle, autoimmune disorders, malaria, latent plasmodium falciparum

## Abstract

Malaria is a global health concern with high morbidity and mortality. It is often attributed to the *Plasmodium (P.) falciparum* species, particularly in sub-Saharan Africa, and it normally has an incubation period of seven to 14 days. Dormant disease secondary to *P. vivax* and *P. ovale* is well-reported, yet only a handful of cases report dormant malaria secondary to *P. falciparum*. Even though malaria is significantly less common in the United States in comparison to other parts of the world, it is still a growing concern given international travel from endemic regions and a growing immunocompromised population. Here, we present a case of *Plasmodium falciparum *malaria in a patient with systemic lupus erythematosus (SLE) with neuromyelitis optica spectrum disorder (NMOSD) and renal transplant without travel to sub-Saharan Africa in 10 years.

## Introduction

Malaria is a complex, multisystem infection with a high level of global morbidity and mortality. In 2020, 241 million cases were reported worldwide, with mortality of 627,000 people [1]. The majority of cases occur in sub-Saharan Africa and South Asia. The five species of malaria that infect humans are *Plasmodium (P.) falciparum*, *P. malariae*, *P. vivax*, *P. ovale*, and *P. knowlesi,* and their primary mode of transmission is the female Anopheles mosquito. Other, less prevalent, modes of transmission include blood product exchange such as transfusions, organ transplants, needle sharing, or congenital transmission [1].

In vector transmission, the Anopheles mosquito must have digested infected blood via a previous blood meal. The mosquito then infects the next human host with sporozoites, which mature into schizonts in liver cells, where they may stay dormant for multiple years. This most commonly occurs with the species *P. vivax* and *P. ovale *but it is rare in *P. falciparum* malaria [2]. Eventually, the liver cells then rupture and release merozoites that infect red blood cells. The cycle repeats within the red blood cell, with trophozoites transitioning to schizonts and being released as merozoites into the bloodstream after red blood cell lysis [3]. This step precipitates symptoms of malaria in the human host, which include cyclic fevers that coincide with the parasites' release into the bloodstream as well as malaise, diaphoresis, weight loss, myalgias, nausea, and vomiting [3,4].

More severe sequelae are cerebral malaria, respiratory failure, acute renal failure, liver dysfunction, and severe anemia. *P. falciparum*, the species most common in sub-Saharan Africa, often causes the most severe infections and contributes to the majority of global malaria deaths. Usually, it has an incubation period of 7-14 days prior to symptomatic disease, and latency is rare [5].

While malaria is much rarer in the United States than in other parts of the globe, approximately 2,000 cases are reported in the United States each year [6]. Most malaria cases in non-endemic regions are introduced via travelers, often those visiting friends and relatives (VFRs), leading to the term “airport malaria” [7]. This spread of malaria is of particular concern in the United States given the ease of international travel and a continued, significant increase in our immunocompromised populations as improved medical management of malignancy, autoimmune conditions, and chronic infections improve life expectancy [8]. Here, we present a case of *Plasmodium falciparum *malaria in a patient with systemic lupus erythematosus (SLE) with neuromyelitis optica spectrum disorder (NMOSD) and renal transplant without travel to sub-Saharan Africa in 10 years.

## Case presentation

A 46-year-old woman with SLE complicated by membranous proliferative glomerulonephritis (MPGN), leading to end-stage renal disease (ESRD) requiring renal transplant and neuromyelitis optica spectrum disorder (NMOSD) presented to a Bronx, New York, hospital after a syncopal episode in the setting of subacute fatigue. She was found to have pancytopenia with white blood cells (WBC) of 2.4 k/μL, hemoglobin (Hgb) of 4.9 g/dL, with a mean corpuscular volume (MCV) of 80.1 fL, and platelet count of 19 k/μL. Bloodwork from an outpatient clinic visit showed the patient had a WBC of 3.8 k/μL, Hgb of 7.5 g/dL, and platelet count of 76 k/μL one week prior. She denied any overt bleeding, menorrhagia, symptoms of active SLE, or acute infection. Her SLE/NMOSD was currently inactive on maintenance rituximab, and she was taking tacrolimus and prednisone for her kidney transplant. She received 2 units of packed red blood cells (pRBCs) and the tacrolimus was quickly tapered. Further studies revealed total bilirubin, direct bilirubin, and haptoglobin were within normal limits, lactate dehydrogenase was mildly elevated at 275 U/L, and the reticulocyte index was low.

**Figure 1:**
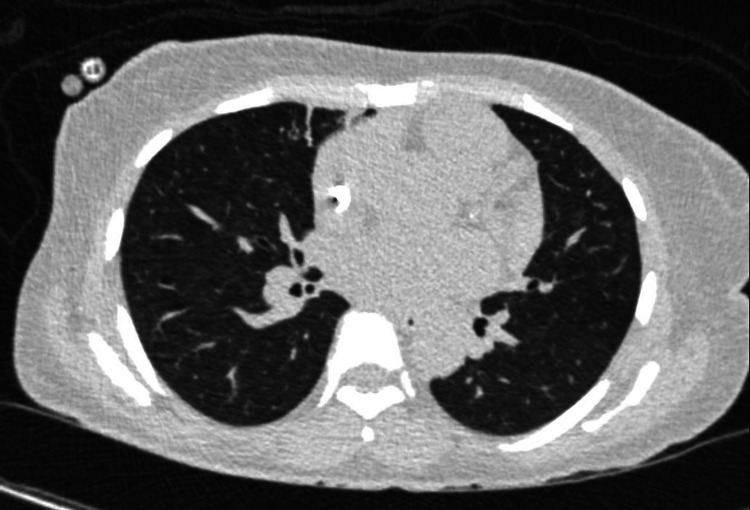
CT Chest CT scan with a focus on branching and ground glass opacity in the right lung apex and the right upper lobe with bronchiectasis

**Figure 2:**
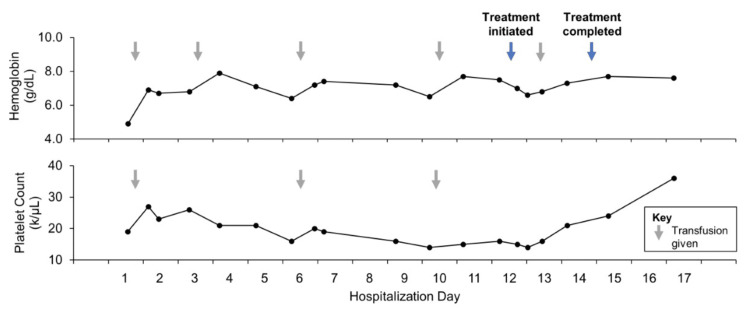
Hemoglobin and platelet count before, during, and after treatment

**Figure 3:**
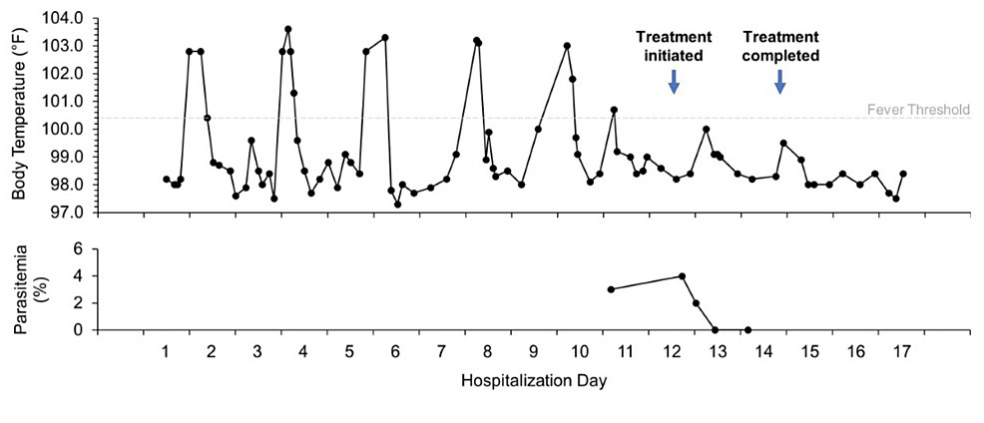
Fever curve and percent parasitemia before, during, and after treatment

Within hours of admission, she developed a fever of 102.8 degrees F without associated chills or diaphoresis and remained hemodynamically stable. Broad-spectrum antibiotics and an infectious workup were initiated. Chest X-ray, urinalysis, urine culture, bacterial and fungal blood cultures, sputum cultures, cryptococcal and Histoplasma antigens, serum fungal markers, respiratory pathogen panel, as well as Epstein Barr and Parvovirus B19 polymerase chain reaction (PCR), HIV antibodies, and PCR were unrevealing. Cytomegalovirus PCR revealed a low viral load of 107 IU/mL. Procalcitonin was >50 ng/mL, C-reactive protein was 20.2 mg/mL, and erythrocyte sedimentation rate was 43 mm/h. Double-stranded DNA was negative and complements 3 and 4 were within normal limits. Chest CT revealed a focus of branching and ground glass opacity seen in the right lung apex and right upper lobe with bronchiectasis (Figure 1). During this time, the fevers continued despite >72 hours on broad-spectrum antibiotics and negative blood cultures Her pancytopenia persisted, requiring transfusions of pRBCs and platelets every two to three days (Figure 2). The patient underwent a bone marrow biopsy, which revealed a hypocellular bone marrow with unremarkable lineages, and a bronchoscopy with bronchiolar lavage, which was also unrevealing. A review of her fever curve revealed the fevers occurred every 48 hours (Figure 3).

Throughout her admission, the patient had multiple complete blood counts (CBCs) due to recurrent transfusion demands. However, on day 10 of hospitalization, a repeat CBC was collected while the patient was febrile to 100.7 degrees F. Later that day, the CBC was flagged for a manual parasite screen by the automated system. Thin film microscopy of a blood smear confirmed the presence of *P. falciparum* with 3% parasitemia as read by the infectious disease department, including an expert in tropical medicine and parasitology, and was sent to the New York State Department for confirmation. A malaria antigen test was also positive. While the patient is originally from Ghana, she had not traveled outside of the United States for approximately 10 years. Also, she has not had any international or out-of-state visitors over the past three years given the COVID-19 pandemic. Furthermore, the patient could not comment on a history of malaria or previous malaria treatment. She received oral artemether-lumefantrine 80-480 mg twice a day for three days. Her cyclical fevers and parasitemia resolved on the second day of treatment (Figure 3). Dormant-stage eliminating medication was forgone.

Outcome and follow-up

The patient was seen in the hematology clinic one week after discharge. Laboratory data from this time revealed her leukopenia had resolved, and her platelet count was improving (now 80 k/μL). Hemoglobin stabilized at 7.7 g/dL without any transfusions and the reticulocyte index at this time was consistent with hyperproliferative bone marrow. Repeat blood smear evaluation with microscopy and malaria antigen testing three months after treatment were both negative.

## Discussion

Here, we present the case of a 46-year-old West African female with a history of SLE and NMOSD requiring renal transplant who presented with pancytopenia and cyclical fevers and was found to have *P. falciparum* malaria without recent travel to or visitors from endemic regions. Latent malaria secondary to *P. ovale* and *P. vivax *is well-documented, with some cases re-emerging after more than a decade of dormancy [9]. However, latent malaria due to *P. falciparum* is much rarer and its reporting is limited to case reports and series. One Canadian case was of a West African male with a history of childhood malaria who presented with *P. falciparum* malaria in the setting of diabetic ketoacidosis two years after his last known travel to West Africa [10]. Dauby’s case series presented three cases of *P. falciparum* malaria with a dormancy range of three months to 10 years. All three patients had immigrated from sub-Saharan Africa and were immunocompromised due to HIV infections or pregnancy [11]. These cases may suggest that the patient’s comorbidities, which modulate their immune system, may have been a trigger for malaria re-emergence. Similar to our patient, who was immunocompromised due to the immunosuppressive regimen for SLE, NMOSD, and renal transplant.

However, another case by Szmitko et. al. presented a healthy young woman without a past medical history who was born in an endemic region of Africa and had a delayed presentation of *P. falciparum* malaria eight years later since travel to an endemic region without an identifiable trigger [12]. In all these scenarios, a common theme is that these patients are immigrants from endemic regions, where they likely had childhood exposure to malaria, which led to immunity. As immigrants from malaria-endemic regions relocate to non-endemic countries, the length and degree of immunity in these individuals have been questioned. Some studies reported immune responses to *P. falciparum* remained, even in individuals whose last exposure was 13-16 years prior [13]. Yet, studies in Sweden suggest immigrants who migrated >15 years prior had a risk of severe malaria similar to malaria-naïve travelers [14]. Others have shown those who immigrated from malaria-endemic regions to non-endemic regions had better clinical outcomes in their first decade in a non-endemic European country and had lower rates of severe malaria in comparison to individuals who had never been in endemic regions [13]. Thus, while the rate of immunity waning in sub-Saharan African immigrants is unclear, there does seem to be data to support at least partial immunity. Thus, these individuals are termed to have “semi-immunity” [13]. This semi-immunity likely played a role in the timing and severity of the case described above.

In the United States, the majority of malaria cases stem from VFRs, like in other non-endemic countries [14]. Our patient denied travel to an endemic region in the past decade and had not hosted visitors from endemic areas in the last three years. Given the COVID-19 pandemic, it is highly probable her last exposure was at least three years prior. While it may be speculated whether this patient was exposed to malaria within her sub-Saharan African community within the Bronx, her high-grade fever without associated symptoms, such as chills, diaphoresis, and headache, hints of a more chronic clinical course. Furthermore, our patient had two additional possible exposures: blood transfusions and her transplanted kidney. As an immunosuppressed patient with ESRD, she had received multiple blood transfusions in the past five years. While blood transfusion-associated malaria is rare, with an estimated incidence of less than one in one million units of blood, it has been reported in the past by the New York Department of Health [15]. Most transfusion-transmitted malaria cases were due to *P. falciparum*, however, reported cases presented within the expected incubation period [15,16]. Our patient’s last blood transfusion was five months prior to her malaria presentation. Similarly, while exceedingly rare in the United States, there are case reports of renal transplant-associated malaria. Like transfusion-related malaria, the majority of transplant-associated infections were due to *P. falciparum* and presented soon after transplant without dormancy [17,18]. Our patient’s presentation of malaria was three years following her renal transplant and further donor information could not be obtained.

Lastly, another unique aspect of this presentation is our patient’s autoimmune history, which added non-infectious inflammatory syndromes to the differential such as Adult Still’s disease and Familial Mediterranean Fever. Given her immunocompromised state and transfusion-dependent state, finding the correct diagnosis in a timely manner was crucial.

In summary, our case highlights individuals with partial immunity to malaria may have a delayed presentation or re-emergence of *P. falciparum* malaria. Despite a lower incidence of malaria in non-endemic countries, malaria must still be considered in a patient with cyclical fevers and a travel history to an endemic malaria region, even if it was remote. Vulnerable populations, such as those on immunosuppressive medications, are at particular risk for these infectious diseases and a delay in diagnosis could have significantly poor outcomes.

## Conclusions

Our report presents a unique case of latent *P. falciparum* malaria in an immunosuppressed individual living in the United States without recent travel or visitors from an endemic region. Partial immunity to malaria from childhood exposure may have played a role in this atypical presentation. Additionally, although less common, *P. falciparum* may be transmitted via blood transfusion or organ transplant. Therefore, cyclical fevers should raise suspicion of malaria in patients who are immunosuppressed or from endemic malaria regions, even in the absence of recent travel to or visitors from endemic regions. The population of patients who travel between endemic and non-endemic malaria regions and those who are immunocompromised is growing. Thus, the number of patients vulnerable to malaria infections is also increasing, and a delay in diagnosis could have detrimental outcomes.
